# Dosimetric Planning Comparison for Left Ventricle Avoidance in Non-small Cell Lung Cancer Radiotherapy

**DOI:** 10.7759/cureus.76543

**Published:** 2024-12-28

**Authors:** Oi-Wai Chau, Stewart Gaede

**Affiliations:** 1 Physics and Engineering, London Regional Cancer Program, London, CAN; 2 Medical Biophysics, Western University, London, CAN; 3 Radiation Oncology, University of California San Francisco, San Francisco, USA

**Keywords:** cardiac substructure, cardiac toxicity, dosimetry, left ventricle, non-small cell lung cancer, radiation-induced cardiac disease, radiotherapy

## Abstract

Introduction: Radiation may unintentionally injure myocardial tissue, potentially leading to radiation-induced cardiac disease (RICD), with the net benefit of non-small cell lung cancer (NSCLC) radiotherapy (RT) due to the proximity of the lung and heart. RTOG-0617 showed a greater reduction in overall survival (OS) comparing higher doses to standard radiation doses in NSCLC RT. V_5Gy_Heart has been reported as an OS predictor in the first- and fifth-year follow-ups. A worsening OS trend was reported in another study where the mean left ventricle dose (mean LV) was ≥14.5 Gy. It is therefore important to spare the heart, specifically the LV, from radiation. Furthermore, dose-limiting factors toward the normal lung should be accounted for to prevent radiation-induced lung injury.

Methods: The LV and left anterior descending artery (LAD) were also contoured on the average four-dimensional computed tomography (4D-CT) dataset that contained clinically defined targets and normal structures for stage III NSCLC RT. The prescribed treatment plans (n=15) were retrospectively optimized with the clinical goals of minimizing the mean LV and mean heart dose while maintaining the dose constraint of V_20Gy_Lung ≤30% and V_95%_PTV ≥95%. Dose-volume histograms were used to compare the heart and lung dosimetric parameters between the delivered and reoptimized RT plans.

Results: A significant reduction (p≤0.044) was observed in the mean LV, mean heart dose, mean LAD dose, max LAD dose, and V_5Gy_Heart from the reoptimized RT plans. V_20Gy_Lung ≤30% and V_95%_PTV ≥95% were maintained, and no differences were observed in the mean lung, V_5Gy_Lung, V_20Gy_Lung, mean esophagus, and max cord.

Conclusion: Minimizing the LV dose in NSCLC RT plans is achievable and dosimetrically advantageous for the heart while maintaining dose constraints to the normal lung and maximizing tumor control. Radiation dose reduction to cardiac substructures may decrease the RICD risk in NSCLC patients.

## Introduction

Lung cancer is the most prominent cancer worldwide (12.4% of total cases) [1]. Radiotherapy (RT) is among the standard curative management for non-small cell lung cancer (NSCLC) patients. V_20Gy_Total Lung is a dosimetric parameter, which defines the percentage of the normal lung receiving a dose of at least 20 Gy. V_20Gy_Total Lung of 30-35% has been reported as the general threshold of an acceptable normal lung dose in RT [2]. Sparing healthy lung tissue from radiation is crucial to prevent radiation-induced lung injury. However, the concern of radiation, which may induce unintentional injury in the myocardial tissue during NSCLC RT, should be addressed, given the proximity of the heart to the target tumor.

Historically, the heart dose in RT planning for NSCLC has not been the primary concern, particularly given that the late toxicities are clinically acceptable for patients with relatively poor prognoses [3]. However, studies have brought attention to the long-term cardiovascular risk associated with high-dose RT. Wang et al. reported that at 8.8 years of follow-up, 23% of stage III NSCLC patients who received dose-escalated RT of 74 Gy across six clinical trials experienced their first cardiovascular event associated with the heart doses in univariable analysis [4]. Furthermore, the RTOG 0617 trial, which compared standard dose (60 Gy) versus high-dose (74 Gy) RT with concurrent chemotherapy for stage III NSCLC [5], found that at a median follow-up of 5.1 years of 496 eligible patients accrued, V_5Gy_Heart, tumor location, radiation dose, and the presence of esophagitis/dysphagia were the factors that affected overall survival (OS) [5]. Moreover, Schytte et al. reported a decline in OS among patients whose mean left ventricle dose (mean LV) exceeded 14.5 Gy up to 12 years post-RT [6]. These findings underscore the importance of minimizing radiation exposure to the heart during RT, specifically the LV from radiation, to mitigate the risk of radiation-related cardiac disease after RT.

Regardless, it is essential to consider the dose-limiting factors towards the normal lung to prevent radiation-induced lung injury (RILI) in patients with lung cancer. Radiation pneumonitis, which is one of the common RILI after thoracic irradiation, has been shown to correlate with worsened OS in patients who had undergone postoperative NSCLC RT [7]. V_20Gy_Total Lung is recognized as a significant predictor of RILI, including pneumonitis and fibrosis, in NSCLC RT [8].

The aim of this study was to perform a dosimetric comparison among NSCLC RT plans to determine whether minimizing the dose to the left ventricle (LV) would result in a significant increase in the dose to other organs at risk, including the normal lung, while ensuring adequate target coverage.

This article was previously presented as a meeting abstract at the 2021 American Association of Physicists in Medicine annual meeting on July 25-29, 2021.

## Materials and methods

The clinically prescribed radiation treatment plans for 15 stage III NSCLC patients, who received a standard 60 Gy in 30 fractions of volumetric modulated arc therapy (VMAT) treatment from 2019 to 2021 with the clinical goal of V_20Gy_Total Lung = 20-30% were retrospectively reoptimized using RayStation7 software (RaySearch Laboratories, Sweden). The study was approved under the Western University Health Sciences Research Ethics Board (123782), and data were accessed from 1 December 2021 to 30 May 2022 for research purposes. The authors did not have access to information that could identify individual participants during or after data collection. The mean age of patients was 70±8 years (range: 66-85). Six of the 15 patients had their tumor located in the right-sided lung, and eight of the 15 patients were male patients.

CT simulation and delineation

Free-breathing four-dimensional computed tomography (4D-CT) simulation was performed on each patient using the Philips Brilliance Big Bore CT scanner (Philips Medical Systems, Cleveland, USA). The patient setup was in the supine position, with scans taken from the upper border of the hyoid bone to the second-to-last thoracic vertebra (T11). 4D-CT scans were then reconstructed into 10 phases using the Respiratory Gating for Scanners (RGSC) system (Varian Medical Systems, Palo Alto, USA). An untagged average 4D-CT dataset (UNTAG AVERAGE) that uses all the projection data acquired during the low-pitch helical CT scan was generated and served as the primary dataset. Additionally, the LV and left anterior descending artery (LAD) were further contoured on the UNTAG AVERAGE CT dataset, which contained all the clinically defined targets and normal structures.

Treatment planning

VMAT planning using RayStation7 software (RaySearch Laboratories, Sweden) was composed of two partial arc 6 MV beams ranging from 300° to 180° in the clockwise and counterclockwise directions. A dose of 60 Gy in 30 fractions was prescribed for each patient, with a minimum of 95% coverage of the tumor planning target volume (D95 PTV). The dose was calculated using collapsed cone convolution.

Dose optimization was performed with the objectives and constraint goals prioritized as follows: (1) heart, (2) total lung, (3) esophagus, (4) spinal cord, (5) brachial plexus, (6) stomach, and (7) remaining normal tissue, according to QUANTEC and dosimetric guidelines in our clinic (see Table 1 for details). Normal lung was considered as total lung volume excluding clinical target volume (CTV) according to the RTOG 0617 definition [5]. For the LV and LAD, no constraint was set due to the lack of literature consensus guidelines available. It was noted that the doses of these two substructures were aimed to be reduced as low as possible.

**Table 1:** Objective and constraint goals for each treatment plan. LV: left ventricle; LAD: left anterior descending artery; PTV: planning target volume.

Objectives
LV and LAD dose: As low as possible
PTV: Dose falls off from 57 Gy to 30 Gy in 1 cm
PTV: Min and uniform dose of 60 Gy
Heart: Max dose of 47 Gy
Stomach: Max dose of 36 Gy
Lung: Max dose to volume 10 Gy to 45%
Constraints
Brachial plexus: Max dose of 60 Gy
Esophagus: Max dose of 47 Gy
Spinal cord: Max dose of 50 Gy
PTV: Min dose to volume 57 Gy to 95%
PTV: Max dose of 62.8 Gy
Total lung: Max mean dose of 20 Gy

Dosimetric assessment

Statistical analyses were performed using IBM SPSS Statistics for Windows, Version 23.0 (Released 2015; IBM Corp., Armonk, New York, USA). Dose-volume histograms (DVHs) and paired t-tests were used to compare the heart and lung dosimetric parameters, including mean heart dose, mean LV, mean LAD dose, V_5Gy_Heart, V_5Gy_Total Lung, V_20Gy_Total Lung, V_20Gy_Right Lung, V_20Gy_Left Lung, in conjunction with mean esophagus and max spinal cord doses, D95 PTV, D1 PTV, and conformity and homogeneity indices among the clinically delivered and reoptimized RT plans.

## Results

**Figure 1:**
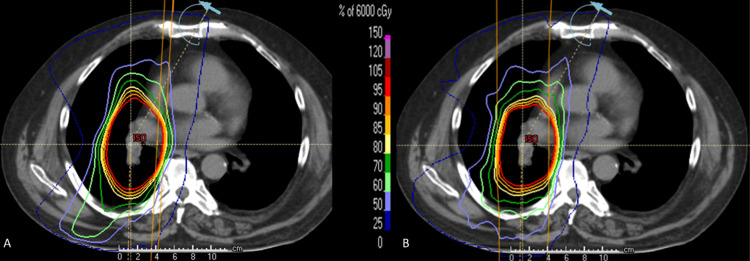
(A) Reoptimized LV sparing treatment plan dose distribution of a representative patient; (B) corresponding clinically prescribed dose distribution. LV: left ventricle.

**Figure 2:**
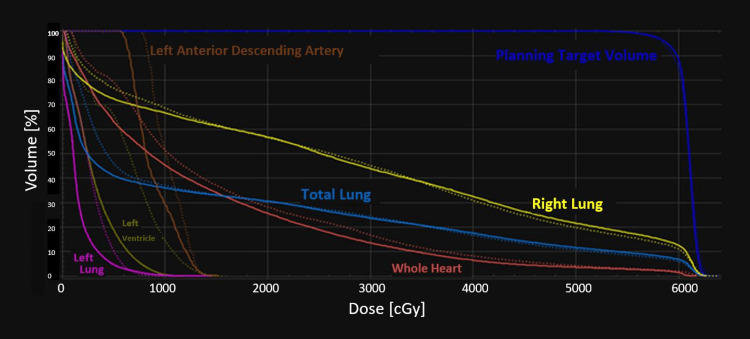
DVH of a representative patient with dose parameters of the reoptimized left ventricle dose sparing treatment plan in sharp lines and clinical prescribed treatment plan in dashed lines. DVH: dose-volume histogram.

**Figure 3:**
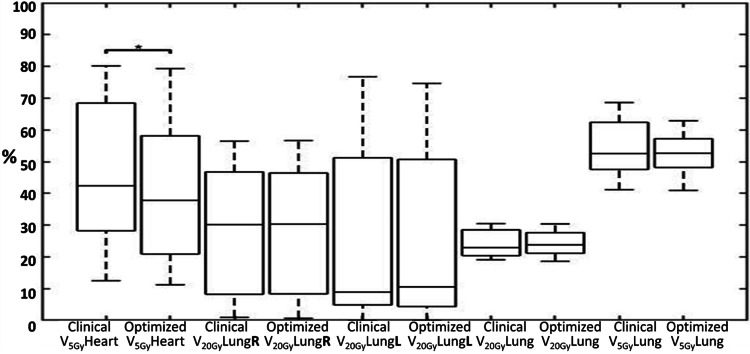
Boxplots (central mark indicates the median) for V5GyHeart, V20GyRight Lung, V20GyLeft Lung, V20GyTotal Lung, and V5GyTotal Lung, determined from the prescribed clinical treatment plans and the reoptimized left ventricle dose sparing treatment plans. A significant reduction (p=0.044) was found only in V5GyHeart. ^*^Significant difference (p<0.05) in paired t-test.

**Figure 4:**
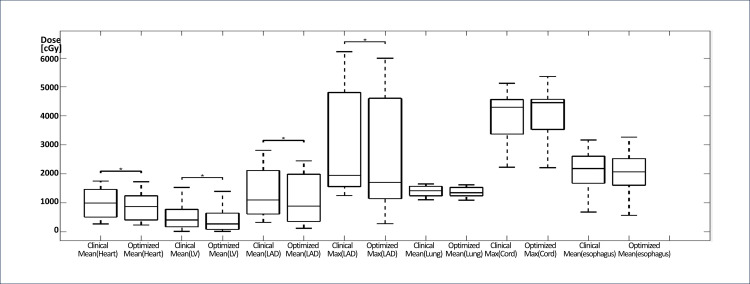
Boxplots (central mark indicates median) for mean heart, mean LV, mean LAD, max LAD, mean lung, max cord, and mean esophagus doses from the prescribed clinical treatment plans and the reoptimized treatment plans. Significant reductions indicated in the figure (p≤0.043) were found in all the dose parameters except for mean lung, max cord, and mean esophagus doses. ^*^Significant difference (p<0.05) in paired t-test.

Significant reductions (p≤0.044) were observed in mean LV, mean heart dose, mean LAD dose, max LAD dose, and V_5Gy_Heart in the reoptimized treatment plans compared to the clinically delivered plans (See Table 2). V_20Gy_Lung ≤30% and V_95%_PTV ≥95% were maintained, and no differences (p ≥0.054) were determined for the parameters of mean lung, V_5Gy_Total Lung, V_20Gy_Right Lung, V_20Gy_Left Lung, V_20Gy_Total Lung, mean esophagus and max spinal cord doses, D95 PTV, and the conformity index. D1 PTV and homogeneity index were significantly different (p≤0.001) compared to the clinically prescribed RT plan but within tolerance (62.32 Gy and 0.942). All of the reoptimized RT plans were clinically feasible and under the literature guidelines (see Figure 1A, B for a representative patient dose distribution, and Figures 2-4 for the DVHs and boxplot results comparing the prescribed clinical and reoptimized treatment plan dose information) [2,6].

**Table 2:** Dosimetry summary, including the heart, the lung, and other normal tissue structures, in conjunction with D95 PTV, D1 PTV, and conformity and homogeneity indices obtained from the prescribed and reoptimized treatment plans of the 15 patients. The p-values obtained from the paired t-tests are also presented. Note all the heart and substructure dose parameters were significantly different between the prescribed clinical and reoptimized treatment plans. Mean LV: mean left ventricle dose; LAD: left anterior descending artery; D95 PTV: 95% coverage of the tumor planning target volume. ^*^Significant difference (p<0.05) in paired t-test. ^# ^Mean values of the 15 patients.

	Prescribed clinical dose parameter^#^	Reoptimized plan dose parameter^#^	p-value
Mean LV (cGy)	587.2	400	0.003^*^
Mean LAD dose (cGy)	1416.7	1139.9	0.028^*^
Max LAD dose (cGy)	3065.5	2698.1	0.009^*^
Mean heart dose (cGy)	1018.3	839.8	0.043^*^
V_5Gy_Heart (%)	48.2	23.9	0.044^*^
Mean lung dose (cGy)	1398.4	1372.1	0.252
V_5Gy_ Total Lung (%)	54.5	52.8	0.147
V_20Gy_ Total Lung (%)	24.1	24	0.854
V_20Gy_ Left Lung (%)	25.6	25	0.219
V_20Gy_ Right Lung (%)	27.6	27.8	0.895
Mean esophagus dose (cGy)	2120.1	2005.3	0.054
Max spinal cord dose (cGy)	3934.6	4027.5	0.894
D95 PTV (cGy)	5781.3	5859.1	0.051
D1 PTV (cGy)	6298.3	6232	0.001^*^
Conformity index	0.966	0.948	0.105
Homogeneity index	0.927	0.942	0.024^*^

## Discussion

Cardiac dose in locally advanced NSCLC RT has not historically been a major concern, as late toxicities are considered acceptable for patients with relatively poor prognoses. Regardless, the RTOG 0617 trial demonstrated that at a median follow-up of two years and five years, cardiac dosimetric parameters of V_5Gy_Heart and V_30Gy_Heart were factors that affected stage III NSCLC OS [5,9]. Additionally, a retrospective analysis of patients with locally advanced NSCLC (stage II to III), who were treated with RT to a median prescription of 66 Gy, with the planning CT heart location recontoured according to the RTOG 0617 guidelines, compared that V_50Gy_Heart <25% versus ≥25% influenced OS [10]. Specifically, the one-year worsened OS rate was 70.2% versus 46.8%, and the two-year OS rate was 45.9% versus 26.7% (p<0.001) [10]. Moreover, it was reported that the median V_50Gy_Heart was significantly higher (20.8% versus 13.9%, p<0.001) for patients with radiation-related cardiac toxicity [10]. Similar findings were noted in other studies, such as Lee et al., which demonstrated an association between V_5Gy_Heart, increased mean heart dose, and higher hazards for acute myocardial infarction in 120 NSCLC patients [11]. Bernard et al. identified V_30Gy_Heart >40% associated with reduced OS in 73 stage IIIb NSCLC patients [12]. Dess et al. reported that mean heart dose and pre-existing cardiac disease were significantly associated with greater cardiac event rates in stage II to III NSCLC patients [13]. In our study, we observed that the dosimetric parameters of the whole heart can be significantly reduced in the LV sparing reoptimized plans, including mean heart dose and V_5Gy_Heart, which is a crucial consideration in light of the worsening OS associations reported by the aforementioned studies [5,9,11].

While the majority of existing studies focus on the whole heart dose in NSCLC, only a limited number have compared dosimetric parameters in cardiac substructures. Turtle et al. [14] compared reoptimized VMAT NSCLC RT plans, achieving significant reductions in the mean heart dose, V_50Gy_Heart, and left atrial wall volume receiving ≥63 Gy (V_63Gy_Left Atrial Wall) in 20 patients with locally advanced NSCLC. A systematic review by Kearney et al. (published 2013-2020) also highlighted the differences in mean heart dose between non-stereotactic ablative radiotherapy (SABR) VMAT and SABR VMAT, with non-SABR VMAT achieving a mean heart dose of 10.5 Gy compared to 6.6 Gy in SABR [15]. In our study, the mean heart dose value (10.18 Gy) extracted from the reoptimized RT plans aligns with the data range reported for non-SABR VMAT by Kearney et al. [15]. However, our analysis extended beyond the whole heart to include critical cardiac substructures, such as LV and LAD, showing a significant reduction of mean LV, mean LAD, and max LAD dose parameters. This is particularly relevant, given that elevated LV and LAD doses were suggested to be associated with a worsened OS and radiation-related cardiac toxicity in stage III NSCLC patients [6,16,17].

It is important to note that the majority of the current literature examining cardiac dosimetric parameters in the context of NSCLC RT has predominantly focused on their relationship with OS instead of the development of cardiac disease itself [5,6,9]. Research linking RT-associated heart disease requires extended follow-up of NSCLC survivors, which includes standard cardiac disease monitoring. As such, the clinical significance or relevance of the role of RT sparing of the heart and substructure for this patient population, while potentially impactful, remains challenging in terms of quantifying long-term cardiac outcomes. Nevertheless, optimizing cardiac dose reduction may play an essential role in improving both survival and quality of life of this patient population.

Limitations

Visualizing the LAD on the 4D-CT datasets is difficult for optimal accuracy of the LAD delineation without the use of intravenous iodine contrast. For future studies, cardiac and substructure atlas with automation can be implemented to increase the efficiency and accuracy of RT planning.

This study tested for the statistical significance of cardiac substructure sparing with an emphasis on VMAT techniques, which aimed to provide NSCLC patients with the optimal VMAT approach, taking into account cardiac sparing and target coverage. It is important to note that in this study, the dose to the cardiac substructures, including the LV and LAD, was aimed to be as low as possible.

In the future, clinical significance must be evaluated to establish a dose-response relationship that can be used to drive future dose optimization objectives (i.e., cardiac substructures). This information may help design new patient-specific treatment strategies that aim to minimize inadvertent heart damage and provide better dose constraint consensus guidelines for better quality NSCLC RT.

## Conclusions

In this study, we demonstrated the clinical feasibility of further minimizing the left ventricle, LAD, and the whole heart dose in NSCLC RT. This was achieved while maintaining the target coverage and sparing other normal tissue, including the normal lung.
